# Cognitive-motor dual task: An effective rehabilitation method in aging-related cognitive impairment

**DOI:** 10.3389/fnagi.2022.1051056

**Published:** 2022-10-18

**Authors:** Xue Tao, Ruifeng Sun, Conglin Han, Weijun Gong

**Affiliations:** ^1^Department of Research, Beijing Rehabilitation Hospital, Capital Medical University, Beijing, China; ^2^Beijing Rehabilitation Medicine Academy, Capital Medical University, Beijing, China; ^3^Rehabilitation Medicine Academy, Weifang Medical University, Weifang, China; ^4^Department of Neurological Rehabilitation, Beijing Rehabilitation Hospital, Capital Medical University, Beijing, China

**Keywords:** aging-related cognitive impairment, cognitive-motor dual task, non-pharmacological interventions, clinical application, mechanisms

## Introduction

With the increase in human life expectancy, the problem of population aging is becoming a growing burden. According to the 2022 World Population Outlook reported by the United Nations, the proportion of people aged 65 or older is expected to grow from 10% in 2022 to 16% in 2050 (United Nations Department of Economic Social Affairs Population Division, 2022). However, the health span or disease-free lifespan has not increased as much as the lifespan (Crimmins, 2015). On average, 16–20% of older adults suffer from aging-related disease or dysfunction (Partridge et al., 2018), which is characterized by a gradual loss of normal physiological function with advancing age. Therefore, human aging-related health problems are the focus and difficulty in the field of medical research. It can damage the perceptual, motor, and cognitive functions, among which cognitive impairment mainly affects patients' activities of daily living and reduces the quality of life (Leuner et al., 2007). At present, there is a lack of effective intervention methods for aging-related cognitive impairment (ARCI), and traditional intervention methods have varying degrees of limitations (van der Steen et al., 2018). Therefore, it is crucial to find an effective intervention method to improve ARCI. In recent years, studies have found that cognitive-motor dual task (CMDT) training exhibits more benefits on ARCI than traditional single-task training (Gheysen et al., 2018; Joubert and Chainay, 2018).

To that end, this paper proposes a look into the definition, clinical features and current intervention of ARCI, the concrete application status, prospect and challenges of CMDT training. These insights allow a more sophisticated understanding of CMDT training and its impact on the ARCI, allowing us to understand how CMDT training compares with other types of interventions in ARCI.

## Aging-related cognitive impairment

Aging is a normal and complex physiological process characterized by a steady decline in various neurophysiological functions, which in turn leads to various physical dysfunctions, including cognitive impairment (Juan and Adlard, 2019). Cognition is the mental activity or process of acquiring knowledge and understanding through thought, experience, and the senses, involving complex information processing, planning, and reasoning (Alchalabi and Prather, 2021). With aging, older adults experience significant declines in cognitive function in terms of processing speed, working memory, and inhibitory control (Hedden and Gabrieli, 2004; Kirova et al., 2015). ARCI may be related to aging in brain structure (gray matter atrophy and reducing, ventricular expansion of white matter, age-related decline in cortical thickness and volume, and constriction of the hippocampus and cerebellum), changes in neurons (neuronal atrophy, spinal narrow, synapse reductions, axon), brain gene expression changes and some conservative biological signaling pathways (Raz et al., 2005; Fjell and Walhovd, 2010; Storsve et al., 2014; Tatti et al., 2016; Bettio et al., 2017). Declines or impairments of resting-state functional connectivity in the superior and middle frontal gyri, posterior cingulate cortex, right insula and inferior parietal lobule are reported to be related to aging (Cera et al., 2019; Li et al., 2021). Although, to some extent, cognitive decline is considered a normal consequence of the aging process, continued progression of cognitive decline inevitably interferes with normal activities of daily living and has the potential to deteriorate into dementia (Harada et al., 2013).

## Current interventions for ARCI

If cognitive impairment in the elderly is left untreated, it may not only worsen to dementia, but may also more severely deprive the elderly of their wellbeing and shorten their life span (Baldwin and Greenwood, 2019). Therefore, we need to actively intervene to prevent and improve ARCI. However, there is a lack of effective interventions for ARCI. Commonly used treatments include pharmacological and non-pharmacological interventions. A large number of clinical studies have been done on pharmacological interventions, however, according to a systematic review of 51 trials, the current evidence is not sufficient to prove that any pharmacological intervention (including dementia drugs, anti-inflammatory drugs, hormones, anti-hypertensives, anti-diabetics, lipid-lowering drugs) is effective in preventing or delaying cognitive decline and improving mild cognitive impairment (Fink et al., 2018). On the contrary, some non-pharmacological interventions such as cognitive training (including traditional cognitive training and computer-assisted cognitive function training), motor training, and non-invasive brain stimulation techniques have shown varying degrees of improvement (Kelly et al., 2014; Hsu et al., 2015; Falck et al., 2019). Unfortunately, all these interventions have limited intervention effects or limited application scenarios. In recent years, many researchers have suggested that cognitive training and motor training may have synergistic effects in improving cognitive function and brain structure (Lauenroth et al., 2016; Wollesen et al., 2020). CMDT training has emerged as an intervention strategy that combines cognitive and motor training. It has been shown that CMDT training may be more effective as an effective combined intervention than a single training modality (Pellegrini-Laplagne et al., 2022).

## Research status of CMDT training as an intervention for ARCI

Traditional cognitive rehabilitation training has many limitations in terms of efficacy and implementation. As a new intervention, CMDT training has shown promising results in the rehabilitation of cognitive disorders of various etiologies. CMDT training has been reported to be more effective in improving cognitive function compared to single training modalities such as traditional cognitive training or motor training (Oswald et al., 2006; Gallou-Guyot et al., 2020b). In stroke survivors with vascular cognitive impairment, the combined intervention produced greater benefits on cognitive function compared to single training (Bo et al., 2019). CMDT training in Parkinson's disease cognitive impairment also significantly improved cognition (Pereira-Pedro et al., 2022). A systematic review by Ali et al. (2022) showed that CMDT training is an effective non-pharmacological intervention to improve global cognitive function in ARCI, particularly in the cognitive domains of executive function, attention, and memory function. Especially in the mild cognitive impairment stage of ARCI, some meta-analyses show that CMDT training is very effective in improving the global cognitive function, memory, executive function, emotion, and other advanced cognitive function and activities of daily living of patients (Law et al., 2014; Zhu et al., 2016). CMDT training was similarly found to have better maintenance of cognitive function in healthy older adults with long-term effects in subjects (Zhu et al., 2016).

There are mainly two classification methods of CMDT training. One is classified according to the intervention mode into simultaneous training (cognitive training and motor training at the same time) and sequential training (cognitive and motor training in sequence, including motor training followed by cognitive training and cognitive training followed by motor training) (Oswald et al., 2006; Legault et al., 2011; Barcelos et al., 2015). The other is to combine different cognitive exercises with one or more different motor exercises, depending on the training content. In general, cognitive training includes different cognitive training elements such as traditional cognitive training, computer-assisted cognitive training, or cognitive function training related to daily life and games (Callisaya et al., 2021; Jardim et al., 2021). Motor training mostly refers to different motor training elements such as aerobic exercise, resistance exercise, or physical and mental exercise (Lauenroth et al., 2016; Li et al., 2022).

There are fewer studies on intervention mechanisms related to CMDT training, and two kinds of intervention mechanisms are generally recognized at present. One is the reciprocal stimulation of neuroplasticity (Wollesen et al., 2020). It has been shown that CMDT training may produce a combined effect of motor training and cognitive training (Eduard Kraft, 2012). Simultaneous performance of both types of training may provide complementary enhancement in terms of increased neurogenesis, synapse formation, promotion of cerebral vascular regeneration, increased blood flow, and enhanced plasticity in the aging brain (Olson et al., 2006; Fabel et al., 2009; Kempermann, 2015). Another view is that cognitive training may be a good guide to the changes in neuronal or brain structure produced by motor training. Motor training is thought to contribute to synaptic plasticity and cell proliferation, while cognitive training guides these newborn neurons into synapses with pre-existing neural networks (Curlik and Shors, 2013; Fissler et al., 2013; Bamidis et al., 2014). The findings suggest that exercise training induces an increase in cerebral blood flow, which in turn increases cerebral metabolic (oxygen, glucose) and neurochemical (dopamine, neurotrophins) activity (Pichierri et al., 2011; Schaefer and Schumacher, 2011). Exercise training also induces the expression of brain-derived neurotrophic factors in the hippocampus, where BDNF enhances brain plasticity by promoting neurogenesis, cell proliferation, and synapse formation in the hippocampus and angiogenesis in other brain regions (Vaynman et al., 2004; Adlard et al., 2005; van Praag, 2009; Wang and Holsinger, 2018). Compared to single exercise training, cognitive training in CMDT training may be more beneficial in translating the exercise-related neurobiochemical and physiological effects into better cognitive performance. (Lauenroth et al., 2016).

## As a good intervention for ARCI, we should know more about CMDT

At present, CMDT training is an important intervention to improve ARCI. A great deal of evidence shows that CMDT training is more effective than single-task training, especially in terms of executive function and attention. The application scenarios and implementation forms of CMDT training are relatively flexible. For ARCI, the application scenarios are mostly community or home scenarios with rich environmental stimulation (Eduard Kraft, 2012). At the same time, more and more studies are devoted to the development of intervention methods that combine games with daily activities, which are suitable for more life-oriented scenarios (Gallou-Guyot et al., 2020a). This approach can not only save medical resources but also help the elderly to achieve better prevention and treatment results in daily life activities and daily living environment. According to the rich environment theory, we suggest that CMDT training should involve multiple cognitive areas and be combined with daily life activities, active social activities, and so on.

Of course, the deficiency of this kind of intervention remains. Firstly, in the current situation of extremely diverse intervention methods, there are relative problems such as insufficient standardization of intervention model, unclear optimal intervention dose, and so on (Zhu et al., 2016; Ali et al., 2022). While developing more and more novel intervention methods, we should further determine the best duration, frequency, intervention type, and combination mode, and formulate more standardized standards (Karssemeijer et al., 2017). Secondly, the results of the current studies are mostly pre- and post-intervention comparisons, lacking follow-up or inconsistent follow-up outcomes. And it is unclear whether the effectiveness of the intervention effect lasts (Callisaya et al., 2021; Li et al., 2022). More cohort studies with higher quality and longer time spans should be designed in the future to explore the long-term effectiveness and outcome reliability of CMDT intervention strategies.

Although CMDT training has been in clinical research for many years, the corresponding intervention mechanism is still in its infancy. In particular, there is a lack of research on the activation of cognitive-related functional brain regions and the underlying neurobiological pathways. The current studies have found that exercise-induced changes in functional brain activation in parietal regions (precuneus, superior and inferior parietal lobule, cingulate gyrus, and posterior cingulate) and associated networks (frontoparietal network, dorsal attention network, and default mode network) may mediate exercise-induced cognition enhancement (Schmitt et al., 2019; Yu et al., 2021). Cognitive training is associated with the frontoparietal network of cognitive control processes, including the posterior parietal cortex and the middle frontal gyrus (Duda and Sweet, 2020). Considering the positive results of a single intervention, CMDT training may be an ideal choice for ARCI (Intzandt et al., 2021). However, there is a lack of research on the relationship between CMDT training and changes in cognitive neuro-brain functional networks. In the future, CMDT training-related neuroimaging measures and molecular marker studies will be important tools to explore the physiological mechanisms underlying this positive effect.

## Concluding remarks

Through the analysis and summary of the literature, we have found that the prevention and treatment of ARCI are facing great challenges in the current society. On this basis, we advocate CMDT training, a multi-domain related rehabilitation strategy, and affirm its effectiveness for ARCI through a literature review. It superimposes and strengthens the effects of the two kinds of training and complements and guides each other, so it is an intervention method worthy of further research and promotion. In addition, we discussed the strengths and weaknesses of dual-task training as an intervention for ARCI, as well as its application prospects and challenges. In a word, for ARCI, CMDT training is a promising intervention method with convenient and effective, low cost, high efficiency, and multi-scene application. The standardization of its clinical application and its internal mechanisms deserve further study.

## Author contributions

XT and WG design this paper. XT, RS, and CH wrote the first draft. WG revised the manuscript. All authors contributed to the conception and design of the work and approved the submitted version.

## Funding

The study was supported by Capital's Funds for Health Improvement and Research (2022-1-2251), the National Natural Science Foundation of China (81972148), and the Natural Science Foundation of Beijing (7222101).

## Conflict of interest

The authors declare that the research was conducted in the absence of any commercial or financial relationships that could be construed as a potential conflict of interest.

## Publisher's note

All claims expressed in this article are solely those of the authors and do not necessarily represent those of their affiliated organizations, or those of the publisher, the editors and the reviewers. Any product that may be evaluated in this article, or claim that may be made by its manufacturer, is not guaranteed or endorsed by the publisher.
